# Miscellaneous

**Published:** 1887-11

**Authors:** 


### MISCELLANEOUS.

Cure for a Bone-felon.—Take the juice of the leaves of rue one tablespoonful; good, strong soft soap, one tablespoonful: and the juice of one red onion; these three articles should be thoroughly mixed, then add a piece of alum and a piece of copperas, each the size of a small marble, finely pulverized; when the whole has been well mixed it is ready for application, by pouring it into a soft, thin leather bag or oil cloth to fit the diseased member, but not very tight, let it remain on till suppuration or scattering takes place. The time it takes this composition to produce suppuration depends on the length of time the felon has been in progress; but it will generally remove the pus from the bone in the course of two hours, when the suffering will cease.

Three Years on Skimmed Milk.—Louis Herbst, who keeps a saloon and hotel on Market street, Camden, and who is one of the best known Germans in South Jersey, celebrated recently his third anniversary of a skimmed milk diet. Mr. Herbst is a large, finely formed man of about 50 years of age, and weighs about 200 pounds. Three years ago he was afflicted with dyspepsia and kidney troubles, and was advised by his phyician to try a diet of skimmed milk exclusively.

He tried the remedy for a couple of months and was so benefited by the diet that he determined to try it for a year. At the expiration of the latter period Mr. Herbst's health was almost perfect. Far from becoming thin or emaciated from the long-continued use of skimmed milk, his form was, if possible, even more rotund than before, and by the advice of his doctor he decided to adopt skimmed milk, as his exclusive diet permanently. For three years he has eaten or drank absolutely nothing but the article named—not even water—and declared to his friends who visited him that he proposed to continue skimmed milk and dispense with steaks, bread and butter and beer for the rest cf his life.—*Philadelphia News.*

Antidotes for Certain Poisons.—A standing antidote for poison by poison oak, ivy, etc., is to take a haudful of quick-lime, dissolve in water, let it stand half an hour, then paint the poisoned parts with it. Three or four applications, it is said, will cure the most aggravated case. Poison from bees, hornets, spider bites, etc., is instantly arrested by the application of equal parts, common salt and bicarbonate of soda, well rubbed in on the place bitten or stung.

Spiritual Legend.—In Dr. Gerson Wolf's work on the Jews of Austro-Hungary, he states, that on the occurrence of death "the mirrors in the dwelling house are either covered over or turned towards the wall and are only re-arranged after the thirty days of deepest mourning have elapsed. The reason given for this is that we should not pander to our vanity during this time of mourning, and that evil spirits which are supposed to lead their dances then should not look at themselves in the glasses. He adds that the water in the house, and sometimes in the next three houses, is poured away, for which the following explanation is given; Samuel the angel of death is a many-eyed creature. He stands at the head of the dying man, and when about to execute his purpose swings forward the sword which he holds in his hands and allows the drop of gall-poison hanging on its point to fall into the mouth of the victim thus putting an end to his life." Coming into contact with the dead renders the sword unclean and therefore the angel of death is obliged to cleanse it. It is feared that he will make use of the nearest vessel containing water, and therefore all stored water is emptied out. . . . During the thirty days of mourning the bed of the deceased remains unoccupied : at its head is placed a burning light because, according to Solomon, "The spirit of man is the candle of the Lord," (Proverbs xx., 27). Also a glass of water and a towel, so that the departed spirit may wash and dry itself. During this time the water evaporates and those who are unacquainted with this law of nature are convinced of the truth of their superstition that the soul of the deceased bathed itself therein.

PROF. LISETTE'S MEMORY DISCOVERY.—Prof. Lisette's new system of memory training, taught by correspondence at 237 Fifth Ave., New York, seems to supply a general want. He has had two classes at Yale of 200 each, 250 at Meriden 300 at Norwich, 100 Columbia Law Students, 400 at Wellesley College, and 400 at Uuiversity of Penn., &c. Such patronage and the endorsement of such men as Mark Twain. Dr. Buckley, Prof. Wm. R. Harper, of Yale, &c., place the claim of Prof. Loisette upon the highest ground.

### HER FIGURE.

Ah, Jesse ! we spied you last eve,
Where the gravel walk winds out of view—
And what said the fair Genevieve,
Is it time to congratulate you ?

Genevieve is as bright as the day,
Her manners, too, are of the best,
There is only one thing in the way,
Her figure, you know, must be guessed.

Her figure, why man 'tis perfection !
Such a neck, such arms, and her bust !
With so many good points for inspection,
You'd surely take some upon trust.

Yes, surely, though "cum grano salis,"
The fashions so stuff and devour.
But the figure I'd now make avail, is
The total of Genevieve's dower.

I must have my dogs and my horses,
My yacht and wine with my grub,
And blushing young maids with long purses,
Is the favorite toast at the club.

DE TRAVERS.

